# Impact of alginate coating combined with free and nanoencapsulated *Carum copticum* essential oil on rainbow trout burgers

**DOI:** 10.1002/fsn3.3192

**Published:** 2022-12-21

**Authors:** Mohammad Hashemi, Shiva Adibi, Mohammad Hojjati, Razie Razavi, Seyyed Mohammad Ali Noori

**Affiliations:** ^1^ Medical Toxicology Research Center Mashhad University of Medical Sciences Mashhad Iran; ^2^ Department of Nutrition, Faculty of Medicine Mashhad University of Medical Sciences Mashhad Iran; ^3^ Department of Food Science and Technology Agricultural Sciences and Natural Resources University of Khuzestan Ahvaz Iran; ^4^ Department of Food Science and Technology Sari Agricultural Sciences and Natural Resources University Sari Mazandaran Iran; ^5^ Toxicology Research Center Medical Basic Sciences Research Institute, Ahvaz Jundishapur University of Medical Sciences Ahvaz Iran; ^6^ Department of Nutrition, School of Allied Medical Sciences Ahvaz Jundishapur University of Medical Sciences Ahvaz Iran

**Keywords:** edible coating, fish burger, nanotechnology, shelf life, solid lipid nanoparticle

## Abstract

*Carum copticum* essential oil (CEO) is known as a valuable active food and pharmaceutical ingredient with antimicrobial and antioxidant activities. Solid lipid nanoparticles incorporated with CEO can overcome their limitations, namely low physicochemical stability and water solubility. In the current study, the antimicrobial and antioxidant activity of free and nanoencapsulated CEO were measured. The results revealed that although the nanoparticles of CEO had higher DPPH radical scavenging activity compared to free CEO, the antimicrobial activity of free CEO toward *Escherichia coli* and *Listeria monocytogenes* was higher than nanoparticles. Fish burger samples coated with free and nanoencapsulated CEO and stored for 12 days at 4°C. Alginate coating without CEO was considered as a control sample. The mean zeta potential, particle size, and polydispersity index (PDI) of nanoparticles were 19.18 ± 0.9 mV, 286.5 ± 18.2 nm, and 0.32 ± 0.01, respectively. The results revealed that lipid oxidation, microbial growth, and production of total volatile basic nitrogen in fish burger samples coated with alginate enriched with nanoencapsulated CEO were lower than free CEO. The main volatile compounds of CEO were *para*‐cymene, γ‐terpinene, and thymol, which were responsible for the antioxidant and antimicrobial activity of CEO. The data obtained by the current study suggest the application of alginate coating with CEO in form of nanoparticle to enhance fish burgers’ shelf life stored at 4°C.

## Graphical abstract

In this study, *Carum copticum* essential oil in the form of solid lipid nanoparticle has been incorporated with alginate coating to increase the shelf life of fish burger. The results indicating the enhancement of the fish burger due to the excellent antioxidant and antimicrobial activity of the nanoencapsulated coating.

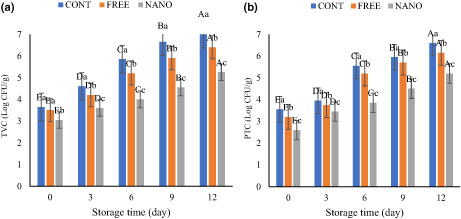

## 1 INTRODUCTION

Food preservatives are vital ingredients to improve the quality and shelf life of foods. Synthetic preservatives like nitrates, butylated hydroxytoluene (BHT), sodium benzoate, butylated hydroxyanisole (BHA), propyl gallate (PG), etc. are widely used because of abundance and low price. The adverse effects of preservatives on human health have been reported and they can increase the risk of several diseases, such as allergies, diabetes, cardiovascular diseases, and cancer (Gómez‐Estaca et al., 2010; Oun et al., 2022). In recent decades, there have been growing worries regarding the use of synthetic preservatives in foods and therefore substantial studies conducted on the reconnaissance of natural preservatives (Carbone et al., 2018; Hussain et al., 2021). Essential oils (EOs) are composed of many valuable natural compounds that play vital roles in human health. EOs are natural preservatives with strong antioxidant, and antimicrobial activities employed as additives in foods, medicine, and cosmetic industries. EOs are generally recognized as safe (GRAS), and extracted from leaves, flowers, seeds, and barks of aromatic plants (Asdagh & Pirsa, 2020).

*Carum copticum* (Ajwain) is commonly known as a medicinal plant with promising pharmacological properties. *Carum copticum* EO and its constituents such as cymene, thymol, and terpinene cause important medicinal properties. Antioxidant, antimicrobial, and antimutagenic activity of *C. copticum* have been reported in the literature (Izadi et al., 2021; Jafarinia et al., 2022; Kazemi, 2015; Maheshwari et al., 2019; Snoussi et al., 2018).

However, EOs are volatile, hydrophilic, and sensitive to light, oxygen, high temperature, and degradation throughout processing and storage (Pandit et al., 2016). Encapsulation, especially solid lipid nanoparticles (SLN), is a good strategy to improve dispersibility, physical and thermal stability, control the release rate of bioactive compounds, and retain the aroma. SLNs are colloidal carrier systems composed of high‐melting‐point lipids such as triglycerides, fatty acids, waxes, hard fats, and partial glycerides, as a solid core coated by surfactants (Kenari & Razavi, 2022; Pandit et al., 2016).

Biodegradable‐based polymers act as a carrier of natural antioxidants and antimicrobials, and protect food from microbial, chemical, and physical corruption (Asdagh & Pirsa, 2020; Esfahani et al., 2022). Alginate is a natural anionic polysaccharide extracted from bacteria and brown algae. Alginate has the potential to encapsulate bioactive compounds with a controlled release (Jafarzadeh et al., 2021; Razavi et al., 2020).

Many studies have recently evaluated the application of different EOs in various edible coatings, such as *Zataria multiflora Bioss* (Hosseini et al., 2013; Sharifi et al., 2017), sage (Ehsani et al., 2020), oregano essential oil (Hosseini et al., 2016; Marzie Kazemi & Rezaei, 2015), *Myristica fragrans* (Kiarsi et al., 2020), and *Bunium persicum* (Sayyari et al., 2021) to improve the shelf life of foods.

Fish burger is one of the secondary minced fish‐based products containing a high amount of proteins, minerals, vitamins, eicosapentaenoic acid, polyunsaturated fatty acids, and docosahexaenoic acid with high bioavailability (Sharifi et al., 2017). The presence of a high concentration of unsaturated lipids is a vital factor that leads to formation of off‐flavors and odors, texture changes, rancid taste, and discoloration (Ehsani et al., 2020).

The edible coatings enriched with natural antioxidants during cold storage can be efficaciously controlled or minimize lipid oxidation and microbial spoilage. Currently, no study was found considering the antimicrobial and antioxidant activity of alginate coating containing *C. copticum* essential oil (CEO) on fish burgers in the literature. The main aim of the research is the application of alginate and essential oil in free and encapsulated forms as a coating in fish burgers. Therefore, (1) the antimicrobial and antioxidant activity of free and nanoencapsulated CEO and (2) the effects of alginate coating incorporated with free and nanoencapsulated CEO on the microbial growth and chemical properties of coated fish burgers during refrigerated storage were evaluated.

## 2 MATERIAL AND METHODS

### 2.1 Materials

All reagents, chemicals, and alginic acid sodium salt from brown algae in the current study were obtained from Sigma‐Aldrich (St. Louis, MO, USA). *Carum copticum* flower was collected in August 2020 from the surrounding areas of the South Khorasan province, Iran. *Carum copticum* was identified by the Department of Botany Sari Agricultural Sciences and Natural Resources University (Sari, Iran).

### 2.2 Preparation of essential oil and gas chromatography analysis

The *C. copticum* flowers were dried in shadow at room temperature and then ground to prepare essential oil. The powdered flowers (100 g) were hydrodistilled by a Clevenger apparatus for 3 h. After that, the anhydrous sodium sulfate was used for removal of residual water from *C. copticum* EO. The EO was kept in a sealed dark bottle at 4°C (Nouri et al., 2021). The CEO was analyzed by gas chromatography–mass spectroscopy (GC6890A and MS5975, Agilent, Palo Alto, USA) equipped with a HP‐5 MS capillary column (30 m^3^ 0.25 mm internal diameter with 0.25 m film thickness as stationary phase) using helium as carrier gas at a flow rate of 0.9 ml/min in a split ratio of 1:20. The volume of sample injection was 0.5 μl. The injector and detector temperatures were adjusted at 240 and 290°C, respectively. The oven temperature was programmed from 50 to 200°C at a rate of 5°C/min and then raised to 240°C at a rate of 10°C/min (Mohammadi et al., 2019).

### 2.3 Formulation of the SLN

The SLN was prepared in accordance with the method previously reported by Laein et al. (2022) with slight modifications. The lipid matrix used in SLN formulation corresponded to 2% w/v of glycerol monostearate as an emulsifier and 0.5% w/v EO. Tween 80 was used as a surfactant at 1% w/v of concentration. The lipid phase melted at 70°C, and then EO at 30.0 mg/ml of concentration was added on hot plate and stirred for 2 min. Then, the water phase was added to the molten lipid matrix with gentle stirring with a magnetic stirrer. The matrix was further dispersed with an Ultra‐Turrax (T‐25, Staufen, Germany) at 13,000 *g* for 5 min to produce the hot primary emulsion. The hot primary emulsion at 70°C was immediately sonicated using an ultrasound bath (D‐7700, Elma, Germany) at 37 kHz for 2 min to produce the nanoparticles. The final SLNs were collected in a container and allowed to recrystallize at room temperature (Laein et al., 2022).

### 2.4 Nanoparticle analysis

The mean particle diameter, polydispersity index (PDI), and surface charge of nanoparticles were measured using a zeta analyzer (Nano‐ZS, Malvern, UK). The nanoparticles (50 μl) were diluted in 1 ml ultrapurified water at neutral pH, then vortexed for 1 min before analysis. The morphological properties of nanoparticles were analyzed with a TEM (JEOL‐JEM 2010) microscope at 200 kV of voltage. The properties of nanoparticles were measured at room temperature.

### 2.5 Antimicrobial and antioxidant activity

The minimal inhibitory concentration (MIC) and minimum bactericidal concentration (MBC) of free and nanoencapsulated CEO against bacterial strains (*Escherichia coli* ATCC 10799 and *Listeria monocytogenes* ATCC 19115) were determined according to Kenari and Razavi (2022). The antioxidant activity of free and encapsulated EO was assessed using DPPH radical scavenging method. Sample solutions were prepared by adding 1 ml of different concentrations of free and encapsulated EO into 4 ml of 0.1 mmol/L of methanolic solution of DPPH and vortexed. The mixture was maintained at room temperature and in dark for 30 min. The absorbance was read at 517 nm. The blank is containing methanol instead of free or encapsulated EO. The antioxidant activity was expressed as a percentage of scavenging activity (Jafarinia et al., 2022; Razavi & Kenari, 2021).

### 2.6 Preparation of coating solution

Alginate solution (3% w/v) was prepared by dissolving 3 g of alginate powder in distilled water, and then 2% glycerol as plasticizer was added and stirred for 30 min at 70°C. The CEO in both free (0.5%) and encapsulated (5%) forms were added to the solution. The mixtures were stirred for 30 min at 40°C to become clear (Sharifi et al., 2017).

### 2.7 Preparation of fish burger and treatments

To prepare rainbow trout burger, 10% wheat/corn flour (2:3), garlic powder (0.2%), sodium chloride (1.2%), sugar (0.6%), and onion powder (0.2%) were added to fresh minced fish meat (87.8%). Fresh rainbow trout fish was purchased from the daily market in Mashhad, Iran, and was immediately transported to the laboratory, and the fish fillet was minced after peeling, deboning, and washing. The fish burger samples were weighed into 50 g portions and were formed into 100 × 8 mm using a burger mold. Coating solutions were sprayed uniformly on the surface of burgers using a compressed high‐pressure, low‐volume air gun device. Both sides of the burgers were treated with respective solutions for 30 s (about 10 ml per burger) and dried for 5 min at 25°C. Fish burgers were packed in polyethylene film of 74 mm thick and then, stored for 12 days at 4°C before analyses (Ehsani et al., 2020; Sáez et al., 2020). Table 1 shows the formulation of different treatments of fish burgers.

**TABLE 1:** Formulation of different fish burger treatments in this study

Treatment	Description
CONT	Fish burger coated with alginate 3%.
FREE	Fish burger coated with alginate 3% and 0.5% CEO in free form.
NANO	Fish burger coated with alginate 3% and 5% CEO in encapsulated form.

Abbreviations: CONT, control; FREE, nonencapsulated essential oil; NANO, nanoencapsulated essential oil.

### 2.8 pH

Briefly, 10 g of each sample was homogenized in 40 ml of distilled water for 2 min. The pH of fish burgers was monitored using a pH meter (HI 99163, Hanna, Romania).

### 2.9 Thiobarbituric acid value

Briefly, 200 mg of the minced fish burger was mixed with 25 ml of butanol. Five milliliter of this solution was mixed with 5 ml of thiobarbituric acid reagent and kept at 95°C for 2 h. After cooling up to room temperature, the absorbance was recorded at 530 nm (Kiarsi et al., 2020).

### 2.10 Microbial analysis

The method described by Sarvinehbaghi et al. (2021) was applied for preparation of serial dilution of fish burgers. Plate count agar using the pour plate method was applied to evaluate total viable count (TVC) and total psychrophilic bacteria (TPC). The inoculated plates were incubated at 37°C for 48 h and 10°C for 7 days, respectively (Sarvinehbaghi et al., 2021). To calculate the *L. monocytogenes* and *Enterobacteriaceae* count, the Palcam agar and violet red bile dextrose agar were used, respectively, and plates were incubated at 30°C for 48 h (Sharifi et al., 2017). *Pseudomonas* count was done using pseudomonas agar after incubation at 25°C for 48 h (Gómez‐Estaca et al., 2010).

### 2.11 Statistical analysis

A two‐way analysis of variance (ANOVA) with Duncan's test (post hoc) and Student's *T*‐test were applied to analyze differences between samples. Differences between mean ± standard deviation were statistically significant at *p* < .05 (95% confidence level). SPSS statistical software (Inc. Chicago, IL, USA) version 20 was used for the statistical analysis.

## 3 RESULTS AND DISCUSSION

### 3.1 Composition of CEO

Essential oils are natural, volatile, and oily liquids, and their great bioactivity has been confirmed. CEO analysis revealed 97.72% of the total compounds, consisting of 19 compounds (Table 2). *Para*‐cymene (34.01%), thymol (29.86%), and γ‐terpinene (29.28%) were the main components of CEO. The data obtained in the current study were in accordance with the previous studies (Jafarinia et al., 2022). Mohammadi et al. (2019) determined the constitute of *C*. *copticum* and detected 25 compounds, and the main constituents were thymol, γ‐terpinene, and *para*‐cymene, respectively (Mohammadi et al., 2019). In other studies, thymol, γ‐terpinene, and *para*‐cymene were also reported as major compounds of *C*. *copticum* essential oil (Goudarzi et al., 2011; Mohagheghzadeh et al., 2007).

**TABLE 2:** Chemical composition of *Carum copticum* essential oil

No	Component	RT (min)	%
1	α‐Thujene	11.35	0.10
2	α‐Pinene	11.74	0.26
3	β‐ Pinene	14.11	1.00
4	Myrcene	14.68	0.54
5	α‐Phellandrene	15.66	0.11
6	α‐Terpinene	16.19	0.50
7	*Para*‐cymene	16.94	34.01
8	Limonene	16.99	0.22
9	β‐Phellandrene	17.07	0.38
10	γ‐Terpinene	18.64	29.28
11	Terpinolene	19.78	0.08
12	Terpinene‐4‐ol	24.84	0.13
13	α‐Terpineol	25.66	0.07
14	Estragole	25.76	0.13
15	Cumin aldehyde	28.04	0.07
16	Carvone	28.34	0.08
17	*E*‐Anethole	30.05	0.74
18	Thymol	30.64	29.86
19	Dill apiol	43.95	0.18
	Totally Identified		97.72

### 3.2 Antibacterial activity

In order to investigate antibacterial properties of *C. copticum* in both free and nanoencapsulated forms, MBC and MIC values were assessed toward *L. monocytogenes* and *E. coli* (Table 3). The higher antibacterial activity was observed in nanoencapsulated form. The higher MIC and MBC of encapsulated form than a pure form of bioactive compounds were also reported by Fazly Bazzaz et al. (2018) for SLN of *Eugenia caryophyllata* EO (Fazly Bazzaz et al., 2018), and Kenari and Razavi (2022) for encapsulated *Bougainvillea spectabilis* extract. Battisti et al. (2021) assessed the antimicrobial effect of micro‐ and nanoparticles of tea tree essential oils. They reported lower MIC and MBC of encapsulated EO compared to pure EO. These results revealed that encapsulation caused the higher antimicrobial activity of essential oil (Battisti et al., 2021). It seems to be that Tween 80 emulsifier can firmly encapsulate CEO and its hydroxyl groups interact with bioactive compounds of essential oil. Also, it might affect the antimicrobial characteristic of nanoencapsulated EO by changing the package state, particle distribution of nanoparticles (Yang et al., 2022), better interaction with microbial cell and SLN, and increasing the solubility and stability of essential oil in aqueous media (Fazly Bazzaz et al., 2018). Jafarinia et al. (2022) reported the most susceptibility of *L. monocytogenes* than *E. coli* for free and encapsulated CEO. They also reported higher antimicrobial activity of encapsulated EO because of its phenolic compounds that disintegrate the cytoplasmic membrane of microorganisms (Jafarinia et al., 2022). In general, gram‐positive bacteria are more susceptible to antibacterial agents in essential oils compared to gram‐negative bacteria. This is due to the outer hydrophilic lipopolysaccharides membrane of gram‐negative bacteria which creates a strong barrier against hydrophobic antimicrobial agents like EOs (Mohammadi et al., 2019).

**TABLE 3:** The MIC and MBC values of free and nanoencapsulated *Carum copticum* essential oil.

Sample	Strain	MIC (mg/ml)	MBC (mg/ml)
NANO	*L. monocytogenes*	0.15 ± 0.05^b^	0.30 ± 0.05^b^
	*E. coli*	0.30 ± 0.05^a^	0.60 ± 0.05^a^
FREE	*L. monocytogenes*	0.05 ± 0.00^c^	0.15 ± 0.00^c^
	*E. coli*	0.15 ± 0.00^b^	0.30 ± 0.05^b^

*Note*: Different lower‐case letters indicate a statistically significant difference (*p* < .05).

Abbreviations: MIC, Minimal inhibitory concentration; MBC, Minimum bactericidal concentration.

### 3.3 Antioxidant activity

Table 4 represents the antioxidant properties of free and encapsulated CEO. The phenolic compounds in the CEO prompt antioxidant activity. Phenolic compounds are widely detected in plants, and a positive relationship between plant polyphenols and antioxidant activity was reported (Kenari et al., 2020; Razavi & Kenari, 2021). The antioxidant activity of free and encapsulated CEO is dependent on concentration. An increase in concentration caused the enhancement of antioxidant activity. These results are in accordance with previous studies that reported that the antioxidant activity of essential oils increased by increasing concentration (Carbone et al., 2018; Kiarsi et al., 2020; Sayyari et al., 2021). In another study conducted by Ghani et al. (2018), the antioxidant activity of cinnamon essential oil nanoemulsions increased by increasing EO (Ghani et al., 2018).

**TABLE 4:** DPPH radical scavenging activity of free and encapsulated *Carum copticum* essential oil (%).

Sample	Concentration (mg/ml)	
	3	6
Ascorbic acid	84.91^Ba^	85.83^Aa^
FREE	17.66^Bc^	27.37^Ac^
NANO	21.45^Bb^	32.12^Ab^

*Note*: Different lower‐case letters indicate a statistically significant difference (*p* < .05). Different upper‐case letters indicate a statistically significant difference (*p* < .05).

### 3.4 Characteristics of nanoparticle (NANO)

The particle size, zeta potential, and PDI are the most important parameters associated with the quality, stability, and other macroscopic properties of nanoparticles. The results of DLS showed that the mean particle size, zeta potential, and PDI of nanoparticles were 286.5 ± 18.2 nm, −19.18 ± 0.9 mV, and 0.32 ± 0.01, respectively. The particle size between 281.5 and 402.2 nm was reported by Hosseini et al. (2013) for thyme EO in chitosan solid nanoparticles, which was in accordance with the results of the current study (Hosseini et al., 2013). Similar particle size reported by Carbone et al. (2018) for solid nanoparticles of *Origanum Hirtum* essential oil was 300.3 nm (Carbone et al., 2018). Zeta potential between −30 and +30 mV exhibited the instability of emulsion systems (Razavi et al., 2020). Therefore, the nanoemulsion must dry quickly to prevent Ostwald ripening and aggregation. The negative zeta potential of nanoparticles could be due to the presence of acidic polyphenol groups. The negative zeta potential of plant essential oil in different coatings was also reported in previous literature (Sayyari et al., 2021). PDI value showed the homogeny of droplet size distribution in emulsion systems, and it ranged from 0 to 1. The PDI of prepared SLN was in the acceptable range for solid nanoparticulated system. Carbone et al. (2018) measured the PDI value of *Lavandula Sumian*, *Rosmarinus Officinalis*, *Thymus capitatus*, and *Origanum Hirtum* EOs in solid lipid nanoparticles between 0.092 and 1 (Carbone et al., 2018). The difference in PDI of nanoparticles depends on preparation method and type of EO. However, the PDI value of lower or equal to 0.3 showed a uniform colloidal system (Chetoni et al., 2016). Nasseri et al. (2016) stated that the particle size of *Zataria multiflora* EO solid lipid nanoparticle was about 255.5 nm. They also reported the PDI and zeta potential as 0.369 and −37.8 mV, respectively, which is in line with the data obtained in our study (Nasseri et al., 2016).

**FIGURE 1:**
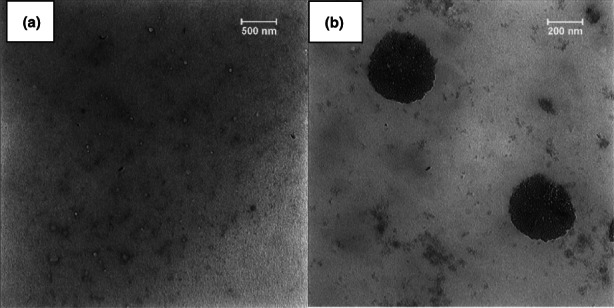
TEM images of CEO loaded in SLN (a) at 500 nm and (b) 200 nm.

The CEO surface morphology loaded in SLN was assessed by transmission electron microscopy at two magnifications (Figure 1a, b). As shown, the obtained SLN is almost spherical and uniform. The size of the particles was around 200 nm in line with the DLS results. A slight difference between DLS and TEM results could be related to different preparation methods, and characteristics of instruments applied for measurement. Correa‐Pacheco et al. (2017) reported well‐distributed and spherical nanoparticles for thyme essential oil in chitosan coating (Correa‐Pacheco et al., 2017). The sphericity of encapsulated EO causes nanoparticles to have an excellent ability for controlled release and protection against encapsulated EO, which is related to the lowest contact area with medium and the longest route for movements of EO (Nasseri et al., 2016).

### 3.5 pH changes

**FIGURE 2:**
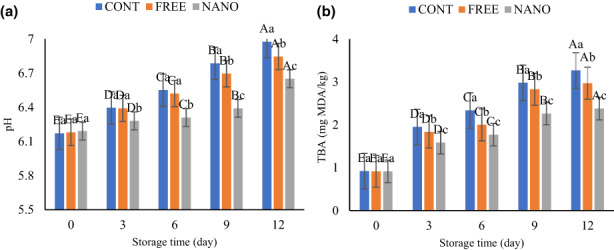
Change in pH (a) and TBA value (b) of fish burgers during storage. Different lower‐ and upper‐case letters indicate statistically significant differences (*p* < .05) between samples on the same day and between days in the same sample, respectively.

The pH values of different fish burgers stored at 4°C were observed in Figure 2a. An increasing trend was observed for all samples related to the volatile basic compounds produced by microbial enzymes and endogenous. The sample NANO showed an acceptable pH value (pH) of <6.5 up to day 12, which was due to the gradual release of phenols during storage. Alginate coating individually did not have an effect on pH values of fish burger samples, while the coatings enriched with EO showed a lower pH than control. These results were in line with other studies which described that the pH value of fish products increased over time while samples coated with a coating containing essential oil showed a lower pH (Karamkhani et al., 2018; Kazemi & Rezaei, 2015; Sayyari et al., 2021).

### 3.6 Thiobarbituric acid (TBA) changes

Lipid oxidation one is of the major chemical reactions that limit the seafood shelf life containing high amounts of polyunsaturated fatty acids. Peroxides as initial lipid oxidation products are unstable and decompose into secondary products. Therefore, estimating TBA value to investigate the extension of lipid oxidation is widely used. TBA values of samples are presented in Figure 2b. The TBA values for fish burgers were increased and significant statistical differences (*p* < 0.05) were observed. The control sample without CEO showed a higher TBA value. Overall, free and encapsulated CEO reduced lipid oxidation of fish burgers. The encapsulated CEO showed a lower TBA value throughout the entire storage time. Sáez et al. (2020) assessed the effects of alginate–chitosan coating embedded with tannins and observed retardation of lipid oxidation in rainbow trout fillets, which was in accordance with our study. Hosseini et al. (2016) reported a lower TBA value in fish fillets coated with gelatin and oregano essential oil (Hosseini et al., 2016). Free radical scavenging and decrement of lipid oxidation may have attributed to the phenolic constituents of EO. Therefore, the gradual release of phenolic compounds from nanoparticles and protective effects of coating in inhibiting oxygen during storage are two important reasons for lower TBA value in fish products coated with edible coatings and natural plan preservatives (Sayyari et al., 2021).

### 3.7 Microbial growth

**FIGURE 3:**
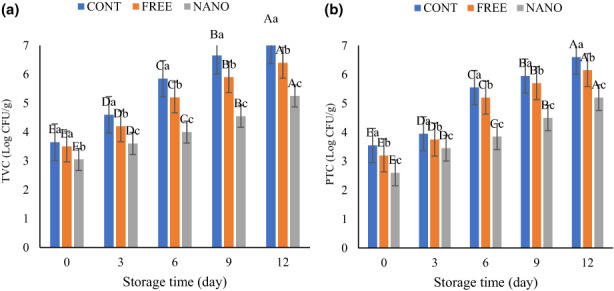
Change in TVC (a) and PTC (b) of fish burgers during storage. Different lower‐ and upper‐case letters indicate statistically significant differences (*p* < .05) between samples on the same day and between days in the same sample, respectively.

The microbial growth results of different fish burger samples are depicted in Figure 3a, b. The primary count of TVC and PTC ranged from 3.05 to 3.65 and 2.60 to 3.55 log CFU/g, respectively, which indicates the acceptable microbial quality of fish burgers. The increasing microbial growth with a statistically significant difference (*p* < .05) was observed during chilled storage. These results are in accordance with Sáez et al. (2020), who stated an increasing trend for PTC of rainbow trout fillet coated with alginate enriched with tannin. Also, they stated that fish fillets without preservatives had higher microbial growth (Sáez et al., 2020).

The fish burger sample without CEO showed a higher TVC and PTC. The efficiency of coating incorporated with CEO in SLN form was higher than coating with free CEO. Nanoencapsulation of essential oil caused the gradual release of phenols from the coating and increase the antimicrobial activity of coating. This was in accordance with the study of Jafarinia et al. (2022) for encapsulated ajowan EO on extending lamb meat shelf life (Jafarinia et al., 2022). Another study by Farahmandfar et al. (2016) revealed an inhibitory effect of TPC on TVC of refrigerated fish surimi during storage (Farahmandfar et al., 2016). As shown in the evaluation of the antimicrobial activity test, the CEO had antimicrobial activity. Therefore, it seems reasonable that the TVC in the fish burger samples coated with free and encapsulated CEO is lower than in control samples due to the antimicrobial constituents in CEO.

**FIGURE 4:**
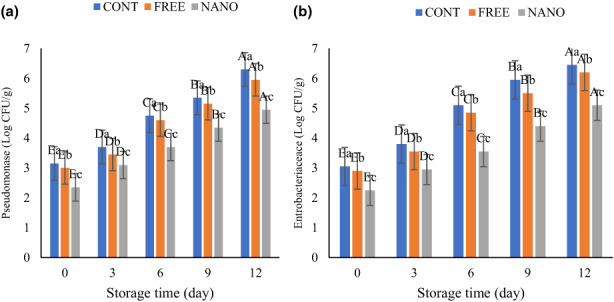
Change in *Pseudomonas* (a) and *Enterobacteria* counts (b) of fish burgers during storage. Different lower‐ and upper‐case letters indicate statistically significant differences (*p* < .05) between samples on the same day and between days in the same sample, respectively.

The initial pseudomonas and enterobacteria of different fish burger samples on the first day of storage time ranged from 2.35 to 3.15, and 2.25 to 3.05 log CU/g, respectively (Figure 4a, b). Similar to TVC and PTC, the count of pseudomonas and enterobacteria significantly increased (*p* < .05) during the storage period. The increasing speed in the control sample was faster, followed by the FREE and NANO, respectively. Kazemi and Rezaei (2015) reported a remarkable development of pseudomonas and enterobacteria in rainbow trout slices without coating compared to samples coated by alginate:gelatin coating enriched with oregano essential oil during storage (Kazemi & Rezaei, 2015).

**FIGURE 5:**
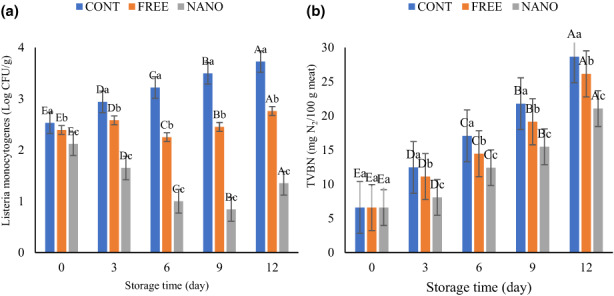
(a) Change in *listeria monocytogenes* count and (b) total volatile basic nitrogen of fish burgers during storage. Different lower‐ and upper‐case letters indicate statistically significant differences (*p* < .05) between samples on the same day and between days in the same sample, respectively.

*Listeria monocytogenes* is one of the challenging foodborne pathogens that generally contaminates seafood products (Sharifi et al., 2017). The results of *L. monocytogenes* growth stored for 12 days at 4°C are shown in Figure 5a. On the first day, the count of *L. monocytogenes* was between 2.12 and 2.39 log CFU/g. It significantly increased in the control sample, while in the samples coated with free and encapsulated EO, the reducing trend was observed up to days 9 and 6, respectively, and then the bacterial counts increased. This result is inconsistent with Sharifi et al. (2017), who reported a significant increase in *L. monocytogenes* count (Sharifi et al., 2017). Gas chromatography–mass spectrograph analysis of CEO showed that the *para*‐cymene, thymol, and γ‐terpinene are the major components of the essential oil. These compounds released from the coating matrix and caused to eliminate bacterial growth. The hydroxyl group increased hydrophilic characteristics of phenolic compounds and accelerated the dissolving of the phenolic compounds in microbial membranes and therefore, eliminating the bacteria.

### 3.8 Total volatile basic nitrogen (TVN)

TVN are amine compounds produced during spoilage of high protein content food products, especially meat. Microbial activities in meat during storage have a major influence on the generation of TVN. The results of TVN value of different fish burger samples during storage time are shown in Figure 5b. The TVN value of all samples increased over time and a statistically significant difference (*p* < .05) was observed. It is related to the bacterial catabolism of amino acids, which is caused to the accumulation of ammonia, different ethylamine, and volatile bases. This result is in agreement with that reported by Hematian et al. (2022) for gelatin film containing *Coleus scutellarioides* to monitor freshness of rainbow fish fillets. They reported increasing rate in TVN during 16 h at 25°C (Hematian et al., 2022). The lower TVN value was observed in burgers coated with nanoencapsulated EO. This was in accordance with the study of Hosseini et al. (2016) who reported TVN of all fish fillets increased during storage. They demonstrated that fish fillets coated with gelatine and oregano essential oil had a lower TVN during storage (Hosseini et al., 2016). Bacteria are responsible for spoilage of aerobically stored fish products at cold temperatures. As seen before, the count of bacteria increased over time, which resulted in more TVN production. The bioactive compounds in the CEO caused disintegrating of the outer membrane of bacteria and released lipopolysaccharides, which indirectly reduce the TVN. The previous study, also reported that applying the plant EOs and extracts due to the preservative effects of phenolic compounds can reduce the TVN value of fish products (Sayyari et al., 2021; Zarei et al., 2015).

## 4 CONCLUSION

In this study, the antimicrobial and antioxidant activity of alginate coating containing free and nanoencapsulated *Carum copticum* essential oil on the extension shelf life of the fish burgers was investigated. The obtained CEO showed suitable antioxidant and antimicrobial properties in the in vitro, and encapsulation caused increase in antioxidant activity of CEO. Both free and encapsulated EO caused potent antimicrobial and antioxidant effects on coated fish burgers. Alginate coating with nanoencapsulated EO exhibited lower lipid oxidation and microbial growth in fish burger, which is related to the gradual release of bioactive compounds from coating during storage. The results of this study suggested a significant potential of alginate coating enriched with nanoencapsulated *Carum copticum* essential oil as a novel method of shelf‐life extension in the food industry in view of increasing concerns in relation to the negative impact of chemical preservatives.

## FUNDING INFORMATION

This study was supported financially by Jundishapur University of Medical Sciences (grant number: U‐98108) and NIMAD.

## CONFLICT OF INTEREST

The authors declare that there is no conflict of interest.

## ETHICS STATEMENT

The current study has been approved by the ethics committee of Ahvaz Jundishapur University of Medical Sciences (IR.AJUMS.REC.1398.433).

## Data Availability

All data are available upon reasonable request.
